# Effect of Transgenic Cotton Expressing Bt Cry1Ac or Cry1Ab/Ac Toxins on Lacewing Larvae Mediated by Herbivorous Insect Pests

**DOI:** 10.3390/plants11202755

**Published:** 2022-10-18

**Authors:** Zheng-Jun Guan, Qiu-Ju Zhou, Hong Shi, Zhi-Xi Tang, Biao Liu, Wei Wei

**Affiliations:** 1 State Key Laboratory of Vegetation and Climate Change, Institute of Botany, Chinese Academy of Sciences, Beijing 100093, China; 2 Department of Life Sciences, Yuncheng University, Yuncheng 044000, China; 3 Institutes of Science and Development, Chinese Academy of Sciences, Beijing 100190, China; 4 Nanjing Institute of Environmental Sciences, Ministry of Ecology and Environment of P. R. China, Nanjing 210042, China

**Keywords:** beet armyworm, cotton bollworm, insect predator, metabolism, non-target effect, trophic level

## Abstract

A simple food chain (plant, insect pests, and predatory arthropods) in an agro-ecosystem was set up here as a model system to elucidate the potential effect of transgenic *Bacillus thuringiensis* (Bt) cotton on non-target organisms. The system included transgenic/non-transgenic cotton, neonate larvae of three herbivorous insects (*Spodoptera exigua*, *Helicoverpa armigera*, and *S. litura*), and predatory lacewing larvae (*Chrysopa* spp.), which represent the first, second, and third trophic levels, respectively. The results showed that transgenic treatments and different densities of prey had significant effects on both body-weight gain of neonate herbivorous larvae and the number of prey captured by lacewing larvae, respectively. It was found that Bt toxin could persist at the third trophic level in lacewing larvae. The diet mixture bioassay showed that body-weight gain of lacewing larvae was significantly affected by various treatments, especially at lower concentrations of plant-expressed Bt toxin in the diet mixture, which caused significant decreases in body-weight gain. In contrast, synthetic Bt toxin at higher concentrations in the diet did not show this effect. Thus, we inferred that Bt toxin indirectly affected the growth of the lacewings and the lacewings may not be susceptible to Bt toxin or are able to metabolize it.

## 1. Introduction

The ecological safety of transgenic crops has been a focus of scientific research and public debate [1,2,3]. Specifically, the effects of transgenic crops with *Bacillus thuringiensis* (Bt) gene on non-target organisms are of great concern in ecological risk assessment [4]. Bt cotton can significantly reduce damage by insect pests [5]; however, Bt toxin expression and insect resistance in transgenic cotton vary in different organs or tissues during different growth periods [6,7,8]. Among the target pests of Bt cotton, cotton bollworm (*Helicoverpa armigera*; CBW) is a main focus in scientific reports. CBW is a dominating insect pest for cotton in fields and its damage has caused heavy losses to agricultural production [9]. With the use of Bt cotton, it was reported that the population of CBW had greatly decreased during the growing season [10]; however, Bt cotton did not offer efficient control of CBW in the late growing season [6,11]. Evolved resistance to Bt cotton has been reported in cotton fields [12,13]. 

Previous studies indicated that Bt cotton can significantly reduce the growth of target pest populations, including body weight, developmental age, pupation, eclosion, behavior, and survival rate [14,15,16]. It was suggested that Bt cotton can provide better control of CBW populations from the first generation to the third generation, but the effect on the fourth generation was relatively poor [17]. The lethality of Bt cotton to CBW is positively correlated with the expression level of Bt insecticidal toxin [16,18]. In addition, the population boom of secondary insect pests has become a critical problem in cotton fields since the commercialized planting of Bt cotton [18,19,20]. Beet armyworm (*Spodoptera exigua*; BAW) is a secondary insect pest in cotton fields and its tolerance to Bt toxin could be generated through laboratory selection [21,22]. Although BAW is not a target pest of Bt cotton, it could become an important pest and much more difficult to control, especially given the widespread application of Bt cotton in recent years [23]. This pest is especially not susceptible to Bt toxin expressed in transgenic cotton and the food source of BAW larvae varies [24]. Resistance of BAW larvae to Bt insecticides has been reported previously [21]. Another pest of the same genus, the cluster caterpillar (*S. litura*; CLC), was also found to be less susceptible to Bt toxin [25]. 

Whether transgenic plants affect natural insect predators has been a hot topic in research and is controversial. Hilbeck and Otto [26] suggested that Bt toxin could negatively affect the growth of predatory lacewing larvae (*Chrysopa cornea*) (Neuroptera: Chrysopidae). Dutton et al. [27] argued that transgenic plants would affect the growth of Lepidopteran insects and, thus, affect the prey quality for lacewings. On the other hand, Bt toxin, expressed in Bt cotton, can be transmitted to higher trophic levels through a non-target pest (e.g., the cotton aphid) and may alter the biology and behavior of a predatory ladybug [28]. In contrast, Tian et al. [29] and Romeis et al. [30] confirmed that Bt toxin did not pose a hazard to the green lacewing, whereas the larvae of both Lepidoptera and cabbage looper (*Trichoplusia ni*) fed on Bt cotton and maize leaves that were exposed to green lacewing larvae (*C. rufilabris*). It was also argued that Bt toxins have no detrimental or adverse effects on natural enemies [31,32,33]. Bt toxin concentrations decreased considerably from one trophic level to the next in the food web, e.g., Wei et al. [34], except for spider mites [35]. However, those studies were performed without considering the effects of insecticides sprays. Lu et al. [36] demonstrated that bio-control services would benefit from the widespread adoption of Bt cotton, as it was assumed that transgenic Bt cotton would boost the amount of prey consumed by lacewing larvae due to the reduced application of insecticides sprays.

Therefore, there is interest in understanding the relationship between the amounts of non-target insects preyed upon by lacewing and the effects of the Bt plant. In this study, we tried to investigate the effect of Bt cotton on insect pests and the preying ability of lacewing larvae in the presence of transgenic plants, to trace the transmission of Bt toxins through trophic levels and to assay the effect of Bt cotton on the predator mediated by herbivorous insect pests.

## 2. Results

### 2.1. Number and Weight of Prey Consumed by Lacewing Larvae 

In bioassay 1, transgenic or non-transgenic cotton treatments (F_1, 50_ = 15.71, *p* = 0.0002) and different densities of prey (F_1, 50_ = 110.82, *p* < 0.0001) had a significant effect on the number of prey captured by lacewing larvae (*C. formosa*); both transgenic cotton treatment of prey and an increase in prey density could boost the number of prey captured by lacewing larvae. In experiment 1, where the density of prey was 10 larvae per cup, the number of prey consumed by lacewing larvae for transgenic Bt and non-transgenic cotton-treated prey was 5.4 ± 0.53 and 4.3 ± 0.59 individuals, respectively. In experiment 2, where the density of BAW larvae was 20 individuals, the number of prey captured by lacewing larvae was 14.3 ± 0.60 and 10.0 ± 0.95 for Bt and non-Bt treatments, respectively. Although on different experimental days, the recorded number of prey consumed by the predator varied (F_4, 50_ = 12.79, *p* < 0.0001), no interaction existed between days and treatments.

In bioassay 2, the number of prey captured by *C. formosa* larvae was different between the two experiments (F_1, 40_ = 5.00, *p* = 0.031) and the two treatments (F_1, 40_ = 30.46, *p* < 0.0001) and among different days (F_4, 40_ = 12.91, *p* < 0.0001). In experiment 1, Bt cotton treatment enhanced the number of prey captured by lacewing larvae from 8.4 ± 0.81 to 11.4 ± 0.81, whereas in experiment 2, the number of prey captured by lacewing larvae for Bt cotton treatment (14.7 ± 0.74) was higher than that for non-Bt-cotton-treated prey (8.7 ± 0.87). In the second experiment, neonate CBW larvae fed actively on cotton disks that were placed at the bottom of the cup after switching of prey from CLC; thus, they were assumed to have been treated by Bt and non-Bt cotton leaves. The change in prey did not alter the number of prey captured by lacewing larvae (F_1, 20_ = 1.31, *p* = 0.27) in experiment 2.

**Figure 1:**
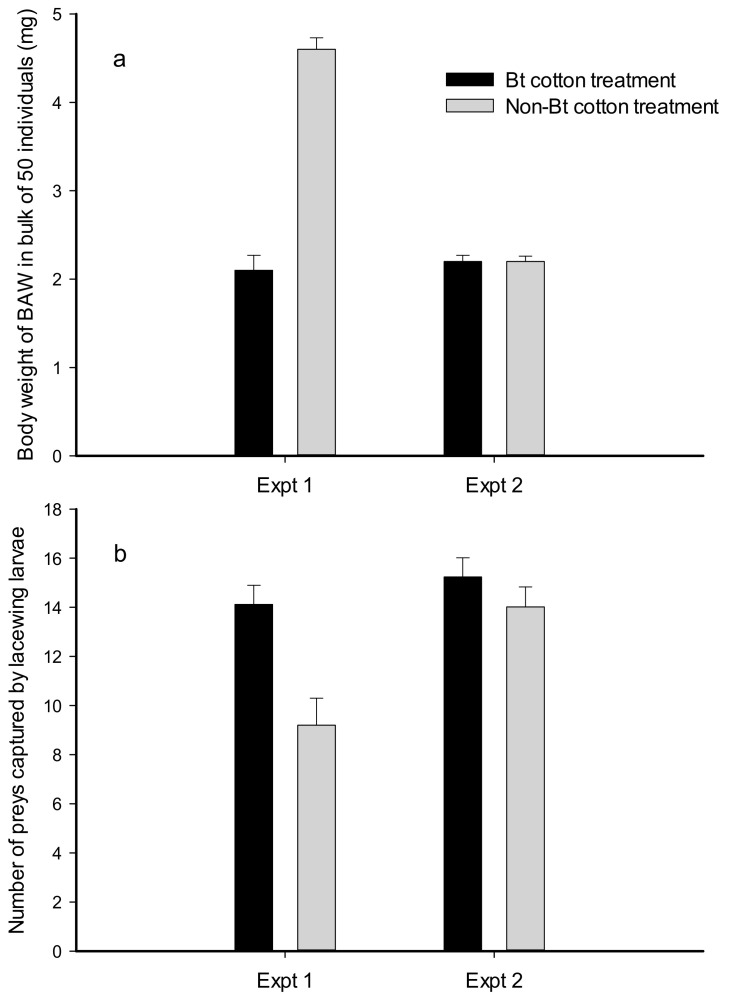
Body weight (**a**) and number (**b**) of neonate beet armyworm (BAW) caterpillars captured by *Chrysopa sinica* larvae. Experiment 1 (Expt 1) included neonate BAW larvae treated for 24 h with transgenic cotton ‘Zhong-30’ leaves and non-transgenic parent ‘Zhong-16’ leaves for Bt and non-Bt treatments, respectively. Experiment 2 (Expt 2) included neonate BAW larvae treated for 24 h with transgenic cotton ‘Zhong-30’ leaves and for 12 h with non-transgenic cotton ‘Zhong-16’ leaves for Bt and non-Bt treatments, respectively.

The results of bioassay 3 are shown in Figure 1. The body weights of neonate BAW larvae were significantly lower for transgenic Bt cotton treatment than non-Bt cotton treatment (F_1, 16_ = 134.73, *p* < 0.0001, Figure 1a) after the same feeding duration of 24 h on cotton leaves in experiment 1. Remarkably, body weights were not different for BAW larvae feeding on transgenic cotton for 24 h or feeding on non-transgenic cotton for 12 h in experiment 2. The number of BAW prey consumed by lacewing larvae (*C. sinica*) was significantly higher when body weight of prey was low, but the numbers were not different than when body weight of prey was similar (Figure 1b) between Bt and non-Bt cotton treatments. This suggested that Bt cotton treatment could reduce the body weight of prey compared to control and stimulate lacewings to prey on more individuals (F_1, 40_ = 11.68, *p* = 0.0013).

### 2.2. Persistence of Cry1Ac Toxin at Trophic Levels

**Figure 2:**
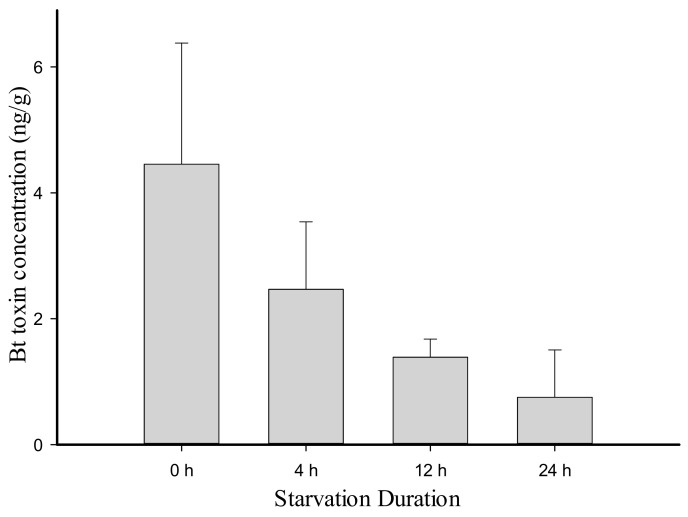
Changes in Bt toxin content in lacewing larvae (*Chrysopa sinica*) bodies during starvation.

Three samples from the 20 transgenic cotton plants (Zhong-30) contained very low concentrations of Bt toxin and, thus, were not used to feed neonate BAW larvae. The average Bt toxin concentration in the remaining 17 cotton plants was 744.6 ± 62.65 ng/g fresh weight (FW), whereas none was detected in Zhong-16. The average Bt toxin content accumulated in the bodies of neonate BAW larvae was 321.7 ± 7.33 ng/g FW (n = 3), which was nearly half of Bt toxin concentration in leaves. Only 4.5 ± 1.93 ng/g FW (n = 8) of Bt toxin persisted at the third trophic level in lacewing larvae when measured right after feeding on BAW larvae was stopped. Bt toxin concentration between these three trophic levels was significantly different (χ^2^ = 20.32, df = 2, *p* < 0.001). Bt toxin content in lacewing bodies declined (Figure 2) during starvation, up to 24 h but the difference was not significant. This implied a great variation in the metabolism of Bt toxin in lacewing larvae.

### 2.3. Effect of Bt Toxin Residual in Larval Body on the Growth of Lacewing Larvae 

No Bt toxin was detected in the leaf samples of Simian-3 (data not shown) and relatively high concentrations of Bt toxins were measured (Table 1) in the third expanded leaves of GK-19 and the results varied among the four times of Bt toxin accumulations (F_3, 47_ = 5.98, *p* = 0.003). Approximately 0.77~19.48 ng/g Bt toxin was detected in the larval powder of BAW that fed on Bt cotton leaves (Table 1), whereas Bt toxin was not detectable in the larval powder of BAW larvae feeding on Simian-3 (data not shown). Thus, no Bt toxin was presented in the diet mixture for “treatment B” in the following feeding test with lacewing larvae. Bt toxins present in larval powder of BAW decreased 80~90% compared to the leaves. Owing to the possible degradation of Bt toxin stored in the freezer, its concentration was very low at the two earlier accumulations. This caused a very low content in the diet mixture fed to lacewing larvae (Table 1).

**Figure 3:**
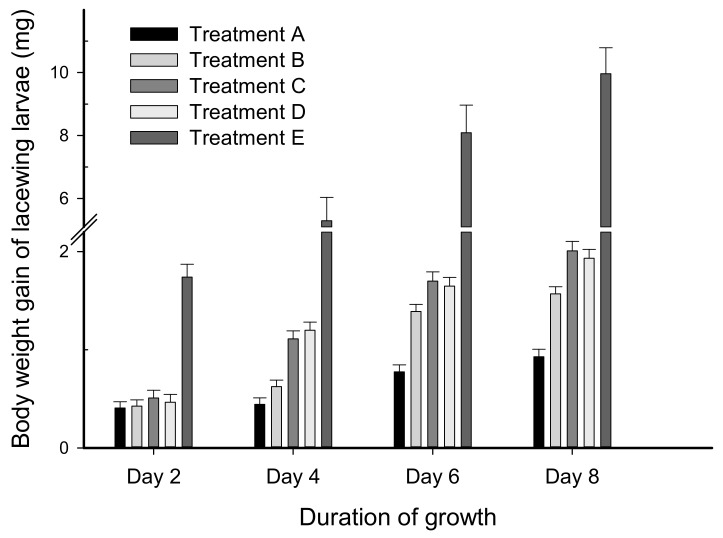
Body-weight gain of lacewing larvae (*Chrysopa sinica*) treated with a synthetic diet plus larval powder of beet armyworm (BAW) fed on transgenic cotton (GK-19) (Treatment A), synthetic diet plus larval powder of BAW fed on non-transgenic cotton (Simian-3) (Treatment B), synthetic diet plus synthetic Bt pro-toxin (Treatment C), blank synthetic diet (Treatment D), and aphids (Treatment E), respectively.

Body-weight gain of lacewing larvae was significantly affected by various treatments (F_3, 322_ = 72.34, *p* < 0.0001). The average weight gain of lacewing larvae in the four synthetic diet treatments ranged from 0.40 to 1.96 mg, whereas the aphid controls ranged from approximately 1.74 to 9.96 mg during the 8 days of the bioassay; the former was obviously lower than the latter (Figure 3). Various treatment durations (i.e., the first, second, third, and fourth 2-day durations) had a significant effect on weight gain of lacewing larvae (F_3, 322_ = 180.98, *p* < 0.0001), and they also had an interaction with the various treatments (F_9, 322_ = 8.54, *p* < 0.0001). Overall, both “treatment A” (feeding synthetic diet plus larval powder of BAW fed on transgenic cotton GK-19) and “treatment B” (feeding on synthetic diet plus larval powder of BAW fed on non-transgenic cotton Simian-3) were significantly different from each other and from other treatments (C and D), whereas no difference was detected between treatment C (treated with synthetic diet plus synthetic Bt pro-toxin) and D (treated with blank synthetic diet only). This result suggested a 5 ng/g concentration of synthetic Bt pro-toxin did not affect the body-weight gain of lacewing larvae, but Bt toxin of lower concentration (Table 1) that was isolated from Bt cotton leaves and was presented in the larval powder could have a negative effect on lacewing larvae. On the fourth and eighth day, the recorded variation between treatments had the same trend with the overall significance, whereas no difference was found among various treatments on the second day, probably due to very low Bt toxin content in the feed. The difference between treatments B, C, and D only bordered on significance.

In contrast to the significant variation in body-weight gain among the various treatments, different treatments did not result in differences in the growth duration from the first instar to the pupae stage or in the actual weight of the lacewing pupae. Treatment A resulted in the longest growth duration of 16.0 ± 0.3 d, while the aphid control (treatment E) resulted in the shortest growth period of 12.8 ± 0.2 d. The pupal weight of the five treatments ranged from 10.4 ± 0.5 to 12.7 ± 0.3 mg, where the weight of the aphid-treated pupae was the lowest since they took the least amount of time (less than 13 days) to reach pupation. Treatments A and C had similar and the lowest pupation rates (57% for A, 55% for C), whereas B and D had the highest rates (73% for B, 70% for D); the aphid-treated larvae (E) exhibited an intermediate rate of pupation (65%). The number of lacewing adults that emerged from pupae was recorded, but their individual dates of eclosion were not included. Although less pupae were obtained for the five treatments, the rate of eclosion was not low; in total, 15, 22, 11, 15, and 12 (male and female) adults emerged from the A, B, C, D, and E treatments, respectively (Table 2). 

## 3. Discussion

While both transgenic Bt and non-transgenic cottons contain secondary metabolites that may adversely affect the growth of insect pest and the integration of foreign Bt gene might alter the metabolite profile in cotton [37,38], our results here showed that the Bt cotton could still provide better control over herbivorous insect pests (e.g., BAW) compared to non-Bt cotton. This study showed that there was a reduced body-weight gain for prey subjected to the Bt cotton treatment and that the number of prey (including CBW, BAW, and CLC) captured by lacewing larvae in transgenic treatments was significantly larger than non-transgenic treatments after 24 h of feeding. However, a feeding duration of 12 h did not result in difference in weight gain; therefore, the number of BAWs captured by lacewing larvae was not affected. The results implied that different feeding durations likely affected the development of prey and eventually changed the number of prey consumed by lacewing larvae. The prey showed growth retardation, weight loss, and small body size after feeding on Bt cotton leaves; thus, the predators had to consume a high number of prey to obtain adequate nutrients [27]. Therefore, the increased number of prey consumed by lacewing larvae might result from the indirect effects of Bt toxin on the nutritional quality of prey, rather than its direct effects. 

It was found that the developmental periods of synthetic diet-treated lacewing larvae were longer than the aphid-treated one. Although the four different synthetic diets showed no difference in the developmental period of the lacewing pupae, the growth period of lacewing larvae in treatment A (synthetic diet plus larval powder of BAW fed on Bt cotton leaves) was the longest, i.e., pupation of the lacewing larvae was delayed and its growth cycle was extended. Both treatments A and C, with artificial diets containing either plant-expressed or artificially synthesized Bt toxin, had lower pupation rates than the other treatments. It was inferred that Bt toxin might have a certain influence on the transformation of larvae into adult lacewings. This specific mechanism of effect needs further study. In addition, the plant-expressed Bt toxin resulted in lower body-weight gain, which was not observed in the synthesized Bt toxin treatment. This might be due to the differences in potential insecticidal activity or protein structure between plant-expressed Bt toxin and synthesized Bt pro-toxin. Although it seems that the predatory function of predators could benefit from reduced growth and weight gain (i.e., smaller body size) of insect pest preys in the field, this advantage could become invalid if predators (e.g., lacewing) are directly or indirectly affected while consuming prey feeding on cotton plants expressing Bt toxins.

In our previous study, we found that Bt toxin residue in insect bodies and feces still had insecticidal ability and could affect other organisms exposed [15]. In this present study, Bt toxin residues at the second and third trophic levels were measured and proved. Although the Bt toxin that persisted in lacewing larvae was low, the finding illustrated that the Bt toxin can pass through the trophic levels through the food chain [34,39], which could cause unintended impacts on the natural ecosystem. The result is consistent with Zhang et al. [28], which indicated that Bt toxin expressed in transgenic cotton can be transmitted to higher trophic level into the coccinellid predator *Propylaea japonica* through a non-target pest insect. Furthermore, in our bioassays, as the starvation time prolonged, Bt toxin content in lacewing bodies declined. During its whole growing period, the lacewing could remove waste from the body only before it begins to pupate [40]. Therefore, it was assumed that Bt toxin may be metabolized inside the lacewing body. This phenomenon was also reported as Bt toxin moves through the food chain involving Bt canola (*Brassica napus*), diamondback moth (*Plutella xylostella*), and lacewing larvae (*C. carnea*) [34]. Although BAW is not the main prey of some predators, such as lacewings, the activity of residual Bt toxin that remained in and/or excreted from their bodies and persisted in the environment may influence susceptible organisms [41,42]. Our results here showed that the body-weight gain as well as pupation rate of lacewings could be indirectly affected by Bt toxin expressed in transgenic plants. Nevertheless, this study did not confirm that Bt toxin has no direct toxicity on predatory enemies of insect pests that feed on Bt plants. Direct toxicity is probably not the only factor that has a lethal effect on natural enemies; other growth indicators should also be considered when performing non-target risk assessment of Bt plants [30,43]. 

Considering the research results presented here, the indirect effects of Bt cotton may be the main factor affecting lacewings. Although Hassanpour et al. [44] showed that lacewing larvae could serve as potential bio-control agents against CBW, Lepidopteran insects are not high-quality prey for lacewings; lacewing larvae had more stunted growth and higher mortality after feeding on Lepidopteran insect larvae compared to other prey species [43,45,46]. In a feeding choice test, lacewing larvae tended to feed on aphids in the field, other than young larvae of Lepidopteran insects [47]. When being attacked by lacewing larvae, young larvae of Lepidopteran insects would fight back; therefore, the lacewings were more likely to become injured and experience higher mortality [26,27]; however, this phenomenon was not observed in the present study due to the limited number of neonate prey larvae used in each assay. 

The results indicated that Bt plants might affect the growth and nutritional quality of herbivorous insects. Owing to low-nutrient foods, the lacewing increased prey numbers. At the end, no significant differences in the various growth indicators were observed between transgenic Bt diet treatment and non-Bt treatment [48]. If the differences in nutrition status among the treatments were eliminated, the amount of variation in prey number of lacewings could disappear. Therefore, the quality and quantity of the prey may be only indirect factors affecting growth of the lacewings. Whether Bt toxin has direct toxic effects on the lacewing (e.g., on body-weight gain and pupation rate) still needs to be further examined.

In general, successful suppression of the targeted insect pests in Bt cotton fields was demonstrated. However, the ecological niches of the suppressed targeted insect pests at the second trophic level could be replaced by secondary or non-targeted insect pests, which could be due to the reduction in broad-spectrum pesticides sprays [19], as well as the reduced presence of bio-control services by natural enemies (including arthropod predators), which resulted from direct or indirect impacts by Bt plants and accumulated Bt toxins in their prey. Impact on the arthropod predators and other natural enemies could affect the fundamental bases of IPM.

Bio-control is the cornerstone of integrated pest management (IPM) and natural pest control methods are normally preferred before using the pesticide options. IPM had called on the strategic integration of multiple control tactics [49], while limiting the dependence on one single technology. By many years of experience using genetically engineered crops, including herbicide-tolerant and/or Bt insecticidal traits, resistance inevitably evolved over time [50,51]. Studies demonstrated the benefits of genetically engineered crops expressing Bt toxins; however, managing resistance evolution of insect pests requires the development of novel resistant crops expressing new and multiple Bt toxins or other resistant traits. Therefore, it is hard to define whether the genetically engineered traits are natural host plant resistances or a series of chemical pesticides upgrading. In addition, while the traditional synthetic sprays of Bt pro-toxin insecticides need to be activated by binding to receptors in the midgut epithelium of the herbivorous larvae to become lethal, Bt toxins directly expressed in plant hosts are already in the activated form and might pose adverse impacts to arthropod natural enemies, even at a very low concentration without the presence of receptors in their digestive tract. Although this unintended effect needs to be confirmed in further studies, the potential adverse impacts on natural bio-control agents could add risk of failure to the IPM strategy, especially if there are additional action modes of Bt toxins on natural bio-control agents [26]. Host plant resistance, including traditional breeding and genetic engineering, could remain a critical component of IPM in the near future [49]; however, diverse approaches and control strategies should be employed for sustainable agricultural production.

## 4. Materials and Methods

### 4.1. Plant and Insect Materials

The plant materials included a Bt cotton (*Gossypium hirsutum*) variety ‘Zhong-30’ containing a *Cry1Ac* gene isolated from *Bacillus thuringiensis* and its non-transgenic parent ‘Zhong-16’; a Bt cotton hybrid ‘GK-19’ containing a recombinant *cry1Ab/1Ac* gene [52] and its conventional and maternal parent ‘Simian-3’. They were planted in a greenhouse at a temperature of 23 ± 2 °C and had light conditions set to a photoperiod of 16L:8D. The third expanded leaves from the apex at the six-leaf developmental stage were sampled for further bioassays. In addition, seeds of broad bean (*Vicia faba*) were sown in humid sand and their seedlings were used to feed aphids (*Aphis craccivora* Koch), the prey of lacewing.

The insect materials, including cotton bollworm (CBW), beet armyworm (BAW), and cluster caterpillar (CLC), and their synthetic diets were purchased from Jiyuan Baiyun Industrial Co. Ltd. (Jiyuan, China). The insects were reared in the insectary at the Institute of Botany at the Chinese Academy of Sciences (IBCAS) (Beijing, China) and the second generation was obtained for the study. The adult lacewings (*Chrysopa formosa*) were captured in the Botanic Garden of IBCAS and reared in the insectary to obtain lacewing larvae for bioassays in this study. Chinese green lacewing larvae (*C. sinica*) were kindly provided by Dr. Fan Zhang at the Plant Protection and Environmental Protection Research Institute, Beijing Agriculture and Forestry Academy of Sciences. All insect materials were kept and reared and the bioassays were conducted in the insectary of IBCAS under appropriate rearing conditions (temperature 25 ± 2 °C, humidity 50 ± 5% and light 16 h: 8 h). Aphids were reared on the seedlings of broad bean and fed to lacewings regularly during the experiment where applicable.

### 4.2. Bioassays on Prey Consumption by Lacewing Larvae and Cry1Ac Toxin Transfer through Trophic Levels

#### 4.2.1. Prey Consumption Bioassays

**Figure 4:**
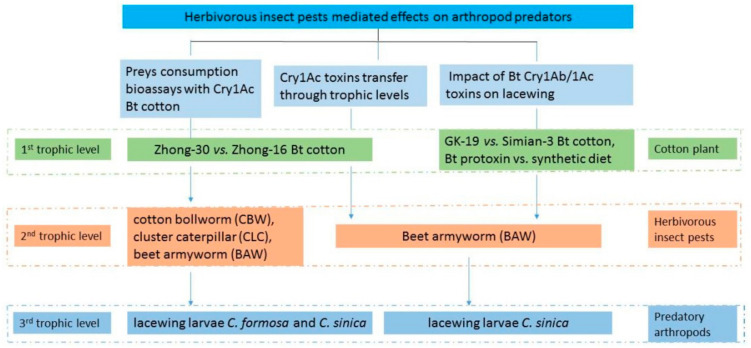
Experimental designs. Bt cotton ‘Zhong-30’ expressing Cry1Ac toxin and its non-transgenic counterpart ‘Zhong-16’ were used to study preys consumption bioassays and to measure toxin transfer through trophic levels, while ‘GK-19’ expressing Cry1Ab/1Ac fusion toxin and its non-transgenic counterpart ‘Simian-3’ were used to study the impact of toxin on lacewing.

Three bioassays were designed to investigate the effect of Bt cotton on the number of caterpillars consumed by predatory lacewing larvae (Figure 4 and Appendix A Table A1). Each bioassay contained two experiments. Lacewing larvae of *C. formosa* were used as predator in both bioassay 1 and 2, while *C. sinica* was used in bioassay 3. Both bioassays used transgenic Bt cotton Zhong-30 and non-transgenic cotton Zhong-16 to feed to herbivorous insect larvae (Figure 4).

In bioassay 1, neonate BAW larvae were fed by leaves of transgenic Bt cotton Zhong-30 and non-transgenic Bt cotton Zhong-16 for 24 h and then they were fed to the third instar larvae of *C. formosa* in plastic cups with permeable covers on the top and 1% agarose at the bottom. The first experiment contained eight and nine replicates for the Bt cotton-treated and non-Bt cotton-treated prey, respectively. Each replicate had 10 neonate BAW larvae and one lacewing larva. The second experiment contained seven and four replicates for the transgenic-treated and non-transgenic-treated prey, respectively. In each replicate of the second experiment, one lacewing larva was placed with 20 neonate BAW larvae, on either a transgenic or non-transgenic cotton leaf disk. 

In bioassay 2, neonate CLC and CBW larvae were allowed to feed for 24 h on the third expanded leaves from the apex of Zhong-30 and Zhong-16, respectively, before they were fed to the third instar larvae of *C. formosa*. In experiment 1, 20 neonate CBW larvae were fed to one lacewing larva in each of the five replicates of both treatments. Experiment 2 contained five and six replicates for non-transgenic and transgenic treatments, respectively. During the first 2 days, 20 individuals of treated CLC larvae were fed to a third instar lacewing larva in each replicate. During the remaining 3 days, the predator was switched to untreated neonate CBW larvae due to a shortage of CLC larvae.

In bioassay 3, neonate BAW larvae were allowed to feed for 12–24 h on the third expanded leaves from the apex of Zhong-30 and Zhong-16, respectively. In experiment 1 of this assay, the larvae were fed for 24 h on both Bt and non-Bt cotton and for 12 h on Zhong-16 in experiment 2. These treated larvae were weighed in a bulk of 50 individuals nine times for each treatment of each experiment and then fed to a third instar larva of *C. sinica* at a density of 20 prey items to one predator. Each treatment of Zhong-16 and Zhong-30 had five replicates in both experiments. 

The number of prey captured by lacewings was recorded in each of 5 days of observation for the three bioassays and the density was maintained at the initial levels by adding an appropriate number of caterpillars.

#### 4.2.2. Detecting Cry1Ac Toxin Transfer through Trophic Levels 

It was suggested that Bt toxin content in the leaves should be assayed in a rearing bioassay [15]. This bioassay intended to study the movement of Bt toxin through a simple food chain (cotton-BAW-lacewing) (Figure 4). Leaf disks of the third expanded leaves were sampled from each of the 20 transgenic Bt cotton plants (Zhong-30) and their Bt concentrations were measured using ELISA. Leaves of non-transgenic cotton plants (Zhong-16) were used as a control. These cotton leaves were used to feed neonate BAW larvae for 24 h; three samples of 50 larvae feeding on ‘Zhong-30’ were randomly sampled to detect Bt toxin accumulation. A sample of 50 larvae that fed on non-Bt cotton was used as a control. These larvae that fed on Bt cotton leaves were transferred to 32 plastic cups with covers; each cup contained 20 larvae and a third instar lacewing larva. One cup contained BAW larvae feeding on non-Bt cotton leaves and a lacewing larva were used as a negative control. The density of BAW larvae in each cup was maintained by adding an appropriate number of larvae. After 3 days of feeding, lacewing larvae were collected and divided into four groups. The first group of eight lacewing larvae was killed immediately by placing into liquid nitrogen individually; the remaining predators were put in cleaned Petri dishes without any food. The second group of ten lacewing larvae was collected after 4 h of starvation. Seven lacewing larvae in each of the third group and the fourth group were collected individually after 12 h and 24 h of starvation, respectively. All lacewing larvae were stored at −80 °C, separately, for further analysis using ELISA Kits (Agdia Inc., Elkhart, IN, USA) to measure the presence of Bt toxin [34]. 

### 4.3. Bioassays on the Effect of Cry1Ab/1Ac Toxins Residual on Lacewing Larvae 

Bt cotton ‘GK-19’ expressing Cry1Ab/1Ac fusion toxin and its non-transgenic counterpart ‘Simian-3’ were used in this experiment (Figure 4). The third expanded leaves of GK-19 at the six-leaf stage were fed to the second instar BAW larvae for 24 h to accumulate Bt toxin at the second trophic levels. Simian-3-fed BAW larvae were used as controls. Bt toxin concentration was measured in the leaves of both GK-19 and Simian-3 using ELISA test that were fed to BAW larvae. Bt toxin in larval body powders was accumulated for bioassay as described in Shi et al. [15]. Ten of the second instar larvae of BAW were added to a Petri dish for each of the twelve and four replicates for GK-19 and Simian-3, respectively. The larvae were collected after 24 h of feeding and stored separately between GK-19 treatment and Simian-3 treatment at −80 °C for >24 h and were ground into dry powder in liquid nitrogen. Part of the larval powder was used to detect Bt toxin concentration and the others was mixed with the synthetic diet [53] of lacewings and fed to the first instar lacewing larvae. The accumulation was repeated four times to generate enough larval powder for further bioassay on the lacewing larvae. Previous research suggests that Bt toxin in synthetic diets persists for 2 days [54]; thus, the mixed diet for the lacewing larvae was re-made and renewed every 2 days and the dry powder that contained Bt toxin obtained at four different accumulations was used in subsequent order to mix with the synthetic diet, respectively. 

Five treatments were set up in this feeding bioassay: (A) 30 replicates of synthetic diet feeding tests with larval powder of BAW that fed on transgenic cotton (GK-19); (B) 30 replicates of synthetic diet feeding tests with larval powder of BAW that fed on non-transgenic cotton (Simian-3); (C) 20 replicates of synthetic diet feeding tests with synthetic Bt pro-toxin (1 mg/mL), and the final concentration of Bt pro-toxin in this treatment was 5 ng/g, which was equal to the reported Bt toxin concentration in larval bodies feeding on GK-19 [15]; (D) 20 replicates of blank synthetic diet feeding tests of lacewings; (E) 20 replicates of aphid feeding tests of lacewing larvae. Each replicate contained one lacewing larva and a certain amount of synthetic diet mixture or aphids. The last treatment was used as a control only and not considered for statistical analysis. 

A piece of squared sponge (1 cm × 1 cm × 1 cm) was placed inside each plastic container (diameter × height = 11 cm × 8.5 cm) with a permeable cover to feed one first instar lacewing larva for each replicate after absorbing enough diet mixture in liquid in each treatment. Body-weight gain of lacewing larvae was recorded every 2 days. The synthetic diet was replaced by aphids after 8 days of bioassay until lacewing larvae pupated. Survival rate of lacewing larvae was also recorded during the bioassay, as well as the rate of pupation, weight of the pupae, and the developmental days of each larval stage. The eclosion rate of lacewing pupae, the sex ratio and oviposition rate of lacewing adults, and hatching rate of lacewing eggs were monitored and recorded to study the possible effects of the diet treatments.

### 4.4. Data Analysis

Statistical analyses were conducted using SPSS 16.0 software. One-way analysis of variance (ANOVA) was used to analyze the variation in Bt toxin content in leaves of transgenic cotton, lacewing bodies and diet mixture, and the number and weight of prey consumed by lacewing larvae. Chi-square test was conducted to test the difference in Bt toxin content at various trophic levels. The GLM (general linear model) procedure was used to analyze the effects of Bt toxin residual on lacewing. Differences in means between treatments were compared using a least significant difference (LSD) test.

## Figures and Tables

**Table 1:** Bt toxin contents in third expanded leaves from the plant apex of GK-19 at the six-leaf stage, in larval powder of beet armyworm (BAW) during the four times of Bt toxin accumulations and in the diet mixture feeding to lacewing larvae.

	Bt Toxin Content in GK-19 Leaves (n = 12) (ng/g Fresh Weight)	Bt Toxin Content in the Powder of Larvae Fed on GK-19 Cotton Leaves (ng/g Fresh Weight)	Bt Toxin Content in Diet Mixture (ng/g Diet Weight)
1	77.4 ± 10.47	0.77	0.004
2	77.8 ± 18.84	1.48	0.016
3	155.5 ± 25.86	19.48	0.418
4	61.1 ± 8.32	11.72	0.251

**Table 2:** Pupation and eclosion of lacewing in the five diet mixture treatments: (A) synthetic diet plus larval powder of beet armyworm (BAW) fed on transgenic cotton (GK-19); (B) synthetic diet plus larval powder of BAW fed on non-transgenic cotton (Simian-3); (C) synthetic diet plus synthetic Bt pro-toxin; (D) blank synthetic diet; and (E) aphid feed.

Treatment	No. of Treated Larvae	No. of Pupae	Eclosion Rate (%)	No. of Adult Males	No. of Adult Females
A	30	17	88.2	10	5
B	30	22	100	10	12
C	20	11	100	7	4
D	20	15	100	7	8
E	20	13	92.3	5	7

## Data Availability

The data presented in this study are available on request from the corresponding author.

## Appendix A

**Table A1:** Bioassays to investigate the effect of Bt cotton on the number of caterpillars consumed by predatory lacewing larvae.

	Expt. No.	Prey (Neonate Larvae)	Predator (3rd Instar Larva)	Prey: Predator Ratio	Predator Feeding Replicates		Preys Pre-Treated Hours	
					Tr.	Non-Tr.	Tr. (Zhong-30)	Non-Tr. (Zhong-16)
Bioassay 1	Expt. 1	BAW	Chrysopa formosa	10:1	8	9	24	24
	Expt. 2			20:1	7	4		
Bioassay 2	Expt. 1	CBW	Chrysopa formosa	20:1	5	5	24	24
	Expt. 2	CLC+CBW			6	5		
Bioassay 3	Expt. 1	BAW	Chrysopa sinica	20:1	5	5	24	24
	Expt. 2						24	12

BAW (beet armyworm): *Spodoptera exigua*; CBW (cotton bollworm): *Helicoverpa armigera*; CLC (cluster caterpillar): *Spodoptera litura*. ‘Zhong-30’ and ‘Zhong-16’ is the transgenic line and the non-transgenic parent cotton, respectively. ‘Tr.’ and ‘Non-Tr’ is the abbreviation for ‘transgenic’ and ‘non-transgenic’, respectively.
